# Editorial for Special Issue “Lipid as a Cancer Therapeutic Target”

**DOI:** 10.3390/ijms22073610

**Published:** 2021-03-31

**Authors:** Elisabetta Albi, Sabine Grösch

**Affiliations:** 1 Department of Pharmaceutical Sciences, University of Perugia, 06100 Perugia, Italy; 2 Institute of Clinical Pharmacology, Faculty of Medicine, Goethe-University Frankfurt, 60590 Frankfurt, Germany

Lipids are structural components of membranes. They act as energy sources and are important for several cellular signaling pathways. They can bind as extracellular ligands at membrane receptors, can be generated as intracellular second messengers, and are important building factors for the environment of various membrane-associated proteins. Huge amounts of lipids have been categorized into main classes and subclasses. Changing the concentration of one lipid does not only lead to changes in this subclass but can also impact the level of other main lipid-classes, which indicates that they are tautly regulated and interconnected. An imbalance in the concentration of different lipids has been associated with cancer and other diseases. Treatments that can reverse these imbalances might be useful therapeutic tools for cancer treatment. Possible treatment targets and drugs that are either in development or are already approved for clinical use are described in four original papers and in four reviews of this special issue. A summary of the lipids addressed in this issue is pictured in Figure 1.

In the first original article of this special issue “Cytosolic Phospholipase A2 Alpha Regulates TLR Signaling and Migration in Metastatic 4T1 Cells”, Tunset, Feuerherm, Selvik, Johansen, and Moestue investigated the influence of a cPLA2α inhibitor X (CIX) on metastasis in murine cells. Using gene expression analysis the TLR and INF-I pathways were identified as new pathways that are affected after cPLA2α inhibition. 

In the article “Long and Very-Long-Chain Ceramides Correlate with A More Aggressive Behavior in Skull Base Chordoma Patients”, La Corte and collaborators studied the ceramide levels in a cohort of patients with a skull base chordoma. The results demonstrate a strong association between total ceramides and dihydroceramides and MIB-1 positive staining as proliferative index. Moreover, long- and very-long-chain ceramides were considered by the authors as molecules related to a prolonged tumor survival and aggressiveness. Reported data induce to identify ceramides as promising tumoral biomarkers in skull base chordomas.

The mutual influence of sphingolipid and proteins/drugs is key in cancer. Given the biological links between sphingolipid and drugs, in the paper “Gentamicin Targets Acid Sphingomyelinase in Cancer: The Case of the Human Gastric Cancer NCI-N87 Cells”, Albi and colleagues investigated the involvement of acid and neutral sphingomyelinase in the response of human gastric cancer cells to gentamicin treatment. The authors demonstrate that gentamicin, a common antibiotic, inhibits gastric cancer cell proliferation with a reduction of cell viability and a decrease of the MIB-1 proliferative index, as well as an upregulation of cyclin-dependent kinase inhibitor 1A and 1B (*CDKN1A* and *CDKN1B*), and growth arrest and DNA-damage 45A (*GADD45A*) genes. Moreover, gentamicin reduces HER2 protein, indicating a minor tumor aggressiveness of the cells. Interestingly, neutral sphingomyelinase is downregulated under the influence of the drug and acid sphingomyelinase is strongly upregulated, indicating the last enzyme as a possible new target of gentamicin in cancer.

Cha and Koo described in “Expression of Autotaxin-Lysophosphatide Singaling-Related Proteins in Breast Cancer with Adipose Stroma” the expression of autotaxin (ATX)-lysophosphatide (LPA) related signaling proteins in breast cancer in dependency of adipose stroma. They related LPA3 expression to disease free survival and overall survival as well CD163-positive macrophages showing that ATX-LPA signaling cascade could be a new target in breast cancer therapy. 

The authors of the review “Cholesterol and Sphingolipid Enriched Lipid Rafts as Therapeutic Targets in Cancer“ discuss in detail the role of lipid rafts located in cell membrane and nuclear membrane in relation to cell fate. After having reported the results of studies showing the importance of each components of lipid rafts as sphingomyelin, ceramide, ceramide-1-phospate, sphingosine-1-phospate, gangliosides, and cholesterol in cancer, the authors describe the changes of lipid rafts paying particular attention in metastasis. Finally, the main papers, showing lipid rafts located in cellular and nuclear membranes as targets of anticancer molecules, are reported. The involvement of lipid rafts in signaling processes modulating proliferation, death, and metastasis is discussed, suggesting that they might be promising targets in cancer therapy.

An overview on mTOR, PI3K, and AKT inhibitors in clinical phase I–III or already approved substances is given in the review article from Hillmann and Fabbro: “PI3K/mTOR Pathway Inhibition: Opportunities in Oncology and Rare Genetic Diseases”. The Pi3K/mTOR signaling cascade is central in the regulation of growth, survival, and proliferation as well as other diseases. Several inhibitors of these pathways are already used for the treatment of neuroendocrine tumors, breast cancer, or tumors of the immune system, and new substances are in prospect for further cancer identities and other diseases. 

As a central player in fatty acid metabolism, the acyl-CoA-synthetases activate free fatty acids to from acyl-CoA that can either be channeled towards anabolic or catabolic pathways. In their review “Targeting Long Chain Acyl-CoA Synthetases for Cancer Therapy”, Sebastiano and Konstantinidou sum up the current knowledge about the expression level of different acyl-CoA synthetase long-chain (ACSL) family isoenzymes in various cancer types and how they are regulated. Furthermore, they give an overview about cancer specific effects of targeting individual ACSL isoenzymes by Triacsin C.

The review “Sphingolipid-Transporting Proteins as Cancer Therapeutic Targets” by Samaha and collaborators shows a wide literature on the relation between sphingolipid transport proteins and cancer with the aim to collect data indicating these specific proteins as molecules useful for innovative pharmacological intervention. The authors report studies on Ceramide Transfer Protein (CERT), Glycolipid Transfer Protein (GLTP), Ceramide-1-Phosphate Transport Protein (CPTP), and Sphingosine-1-Phosphate Transporters. Despite some opposite opinions, CERT is the most studied protein as pharmacological target in cancers resistant to chemotherapy. CERT is able to free ceramide from endoplasmic reticulum. HPA-12, a small-molecule inhibitor of CERT, elevates reticulum endoplasmic stress reducing CERT-mediated clearance of ceramide. The sensitization of cells to chemotherapy is obtained with CERT depletion with consequently decreased ceramide transport and increased levels of ceramides.

## Figures and Tables

**Figure 1:**
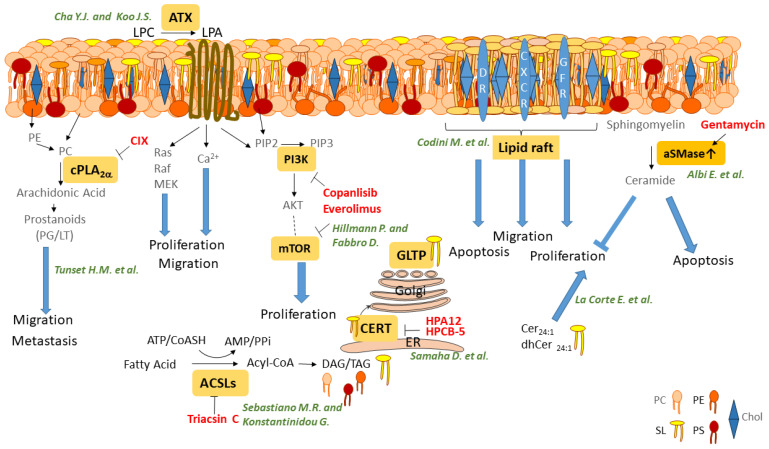
Summary of the different lipid classes and their metabolizing enzymes that are addressed as targets for cancer therapy in this special issue. ACSL; Acyl-CoA Synthetase Long-chain, ATX; Autotaxin, CERT; Ceramide Transport Protein, Chol; Cholesterol, GLTP; Glycolipid Transfer Protein, LPA; Lysophosphatide, cPLA2α, cytosolic Phopholipase A2 alpha, PG; Prostaglandin, LT; Leukotrien, IP3; Inositol 3,4,5 triphosphat, DAG; Diacylglycerol, TAG; Triacylglycerol, CIX; cPLA2a inhibitor X, PC; Phosphatidylcholine, PE; Phosphatidyl-Ethanolamine, PS; Phosphatidylserin, Sl; Sphingolipid, Chol; Cholesterol, aSMase; acid Sphingomyelinase, DR; Death Receptor, GFR; Growth Factor Receptor, CXCR; C-X-C motif Chemokine Receptor.

